# Surface alloying of FeCoCrNiMn particles on Inconel-718 using plasma-transferred arc technique: microstructure and wear characteristics

**DOI:** 10.1039/d1ra03778a

**Published:** 2021-08-20

**Authors:** N. Jeyaprakash, Che-Hua Yang, G. Prabu, K. Ganesa Balamurugan

**Affiliations:** Graduate Institute of Manufacturing Technology, National Taipei University of Technology Taipei 10608 Taiwan Republic of China prakash84gct@gmail.com prakash@ntut.edu.tw chyang@ntut.edu.tw +886-2-2731-7191 +886-2-2771-2171, ext. 4512; Additive Manufacturing Center for Mass Customization Production, National Taipei University of Technology Taipei 10608 Taiwan Republic of China; Department of Production Engineering, National Institute of Technology Tiruchirappalli 620015 Tamil Nadu India; Department of Mechanical Engineering, IFET College of Engineering Villupuram 605108 Tamil Nadu India mgprabu@gmail.com gbmpondy@gmail.com

## Abstract

Inconel-718 (IN-718) is a commonly used nickel-based superalloy in various fields, such as gas turbine and power generation applications. However, the lower wear and oxidation resistance hinder their wide usage. In this work, FeCoCrNiMn particles were mechanically ball-milled and preplaced on the IN-718 substrate. Then, the preplaced FeCoCrNiMn particles were scanned by heat source using plasma-transferred arc (PTA) technique. The effect of PTA alloying on the phase changes, microstructure, nanohardness and wear resistance has been investigated. The result showed that the PTA region contained different phases, such as FCC, BCC and intermetallic. No cracks were observed in the PTA alloyed region. Moreover, the porous free structure was viewed in the alloyed region, which revealed that the PTA alloying process was effectively used to perform the alloying process. More hard phases, such as NiFe, CoMn, Cr_9_Mn_25_Ni_21_, MnNi, FeCo, FeMn and MnCo, were formed on the PTA-alloyed region. The obtained wear rate of the substrate specimen at 30 N applied load is 2.45 × 10^−3^ mm^3^ m^−1^ and 1.79 × 10^−3^ mm^3^ m^−1^ for the PTA specimen. Similarly, the wear rate of the substrate specimen at 50 N is 5.38 × 10^−3^ mm^3^ m^−1^ and for the PTA sample, it is 2.29 × 10^−3^ mm^3^ m^−1^. The PTA specimen showed lower CoF than the substrate specimen due to increased surface hardness and minimum deformation of asperities. The primary wear type was mildly abrasive, accompanied by slight oxidative wear. Oxygen reacted with the surface alloying elements and formed different oxides, such as CoO, Cr_2_O_3_, MnO_2_, Mn_2_O_3_, Mn_3_O_4_, FeO and Fe_2_O_3_. These dense oxidation films covered the working surface and enhanced the wear resistance. The worn-out PTA surface showed that the wear scar depths were shallow and lower than the substrate, and reduced the roughness.

## Abstract 2

Inconel-718 (IN-718) is a commonly used nickel-based superalloy in various fields, such as gas turbine and power generation applications.

## Graphical abstract

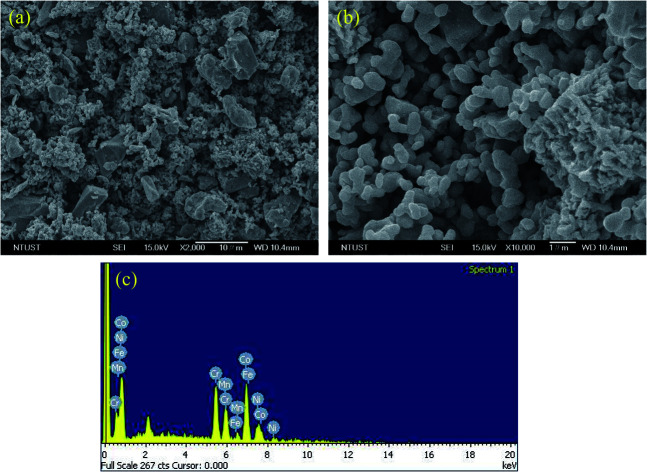

## 1. Introduction

Superalloys are the specific metal alloy group that can withstand high temperature environments. Resistance to creep and oxidation are the primary design criteria for these alloys. Iron, cobalt or nickel-based superalloys are used in aero-engine applications.^1^ Among the superalloys group, nickel-based superalloys are the best suited for turbine engine materials due to their high strength and long fatigue life. Besides, the nickel-based superalloys are thermally stable due to the lowest coefficient of thermal expansion.^2^ Among these, Inconel-718 is a nickel–chromium–iron based superalloy, which has specific properties such as high strength, low thermal conductivity, high resistance to thermal fatigue, corrosion and creep resistance.^3^ In turbine applications, nickel-based superalloys experience fretting fatigue failure due to wear and cyclic contact stress.^4^ Generally, wear provides an adverse effect on the metallic materials performance, and is still challenging in many industries.^5^ In recent years, researchers worldwide have been attempting various methods and materials to overcome this constraint on metallic materials. Surface modification is the widely utilized method to protect and improve the wear resistance of the metallic material surface. Among the different surface modification techniques, the plasma transferred arc (PTA) alloying is an efficient method due to its notable advantages, such as excellent arc stability, short dilution rate of substrate and higher temperature generation during the alloying process.

Khan *et al.*^6^ performed NiCr–Cr_2_O_3_ and Al_2_O_3_ + 40% TiO_2_ plasma-sprayed coating on a nickel-based superalloy, and conducted dry sliding wear tests. Results revealed that Al_2_O_3_ + 40% TiO_2_ showed improved wear resistance compared to NiCr–Cr_2_O_3_ on the nickel-based superalloy. Sharma *et al.*^7^ studied the effect of electron surface melting on Inconel-718 with different combinations of beam current and scan speed. Results showed that the electron beam melting improves the wear resistance and hardness. Chun *et al.*^8^ deposited WC-metal powder on the Inconel-718 alloy through High Velocity Oxygen Fuel (HVOF) thermal spraying. Results indicated that binder materials like Cr and Ni were completely melted and bonded with WC, and hard carbides were formed on the WC-metal coating. From the above literature, most conventional materials and techniques have been attempted to improve the wear resistance of the nickel-based superalloys. Still, new material combinations and methodologies are in search by the researchers to upgrade the surface modification of the nickel-based superalloys.

In recent years, the combination of five or more elements has been extensively studied and utilized in various industries.^9–13^ These alloying elements exhibit outstanding surface properties, like high corrosion resistance, wear resistance, microhardness and oxidation resistance. Chaorun Si *et al.*^14^ deposited FeMoCrCo particles on a stainless steel substrate using flame spraying techniques. Microhardness of the coating reached around 981.6 HV_0.1_, which was much higher than that of the stainless steel substrate material. The wear behaviour of the coating was tested at 19.6 N normal load condition, and the wear rate was measured to be 1.56 × 10^−5^ mm^3^ Nm^−1^ with the Coefficient of Friction (CoF) of 0.265. The CoF was relatively steady throughout the tribological test. Qingfeng Ye *et al.*^15^ studied the corrosion behaviour of the CrMnFeCoNi coating on the A36 steel substrate through laser surface alloying technique. Potentiodynamic polarization tests at 0.5 M H_2_SO_4_ and 3.5 wt% NaCl were studied, and it was found that the laser alloyed surface exhibited superior corrosion resistance with one-magnitude lesser *i*_corr_ value than that of the A36 steel substrate. Thomas Lindner *et al.*^16^ produced the CrFeCoNi and CrMnFeCoNi bulk material using arc-melting techniques and conducted tribological tests. Results showed that the fcc-phase was formed, and improved the microhardness and wear resistance. Gnyusov *et al.*^17^ effectively fabricated the FeCrVMoC coating on a steel substrate using the PTA alloying process. Results revealed that the maximum wear resistance was obtained in the PTA processed eutectic steel compared to the substrate material. Xinke Deng^18^ prepared the wear-resistant Mo–Fe coating on the AISI 1045 steel substrate by PTA technique. Pure Mo powders were used as the precursor material during the alloying process. The MoO_3_ oxide layer was formed on the worn-out surface that had a superior friction reducing property, and contributed toward increasing the wear resistance of the Mo–Fe coating. The outcome of experimentation showed that the wear resistance of the Mo–Fe coating was much higher than that of the steel substrate.

Still, there is a lack of literature to understand the effect of this system on the wear resistance of the nickel-based superalloys. Similarly, plasma transferred arc (PTA) has not been extensively utilized for surface modification of Inconel-718. Using the FeCoCrNiMn as a coating material along with the PTA alloying process will not only widen its application, but also reduce the cost. Besides, investigation of the PTA alloying of the FeCoCrNiMn coating on the Inconel-718 substrate has not been carried out in previous studies. Moreover, the nanohardness and wear behaviour at different load parameters still remain unknown. Hence, in the present study, the FeCoCrNiMn coating is prepared on the Inconel-718 surface, and the alloying process is performed using the PTA technique. Furthermore, the phase formation, microstructure, nanohardness and wear behaviour of PTA alloyed specimens are investigated and their corresponding roughness values reported.

## 2. Experimental procedure

### 2.1 Starting materials

**Fig. 1:**
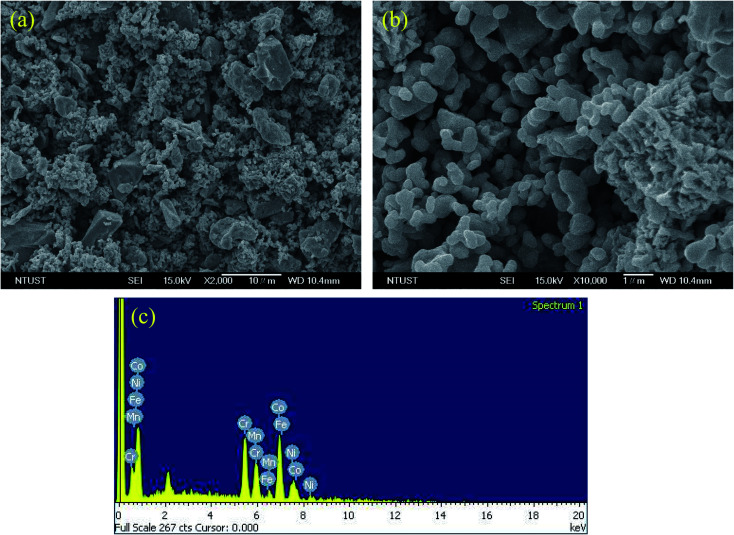
(a) FESEM picture of mechanically ball-milled powder, (b) magnified view of (a), and their corresponding elemental analysis graph (c).

Nickel-based superalloy Inconel-718 was used as the substrate material; the chemical composition of Inconel-718 is shown in Table 1. The substrate samples were prepared by cutting the material into 40 × 40 × 15 mm dimensions. Then, the substrate samples were sand-blasted to obtain a surface roughness of ∼10 μm to provide better bonding between the coating material and the substrate. Furthermore, the elements Fe, Co, Cr, Ni and Mn were mixed with an equal molar ratio and conventionally ball-milled for 12 hours. The powder-to-ball ratio, speed and particle size of the powder were 1 : 15, 350 rpm and 5–10 μm, respectively. Fig. 1(a–c) shows the FESEM image of the ball-milled powder morphology and the corresponding elemental analysis.

**Table 1:** Chemical composition of Inconel-718 alloy

Elements	Ni	Cr	Nb	Mo	Co	Al	C	Fe
Wt (%)	52.50	19.04	4.95	3.10	1.02	0.60	0.08	Bal.

### 2.2 Preplaced coating and plasma transferred arc (PTA) processing

**Fig. 2:**
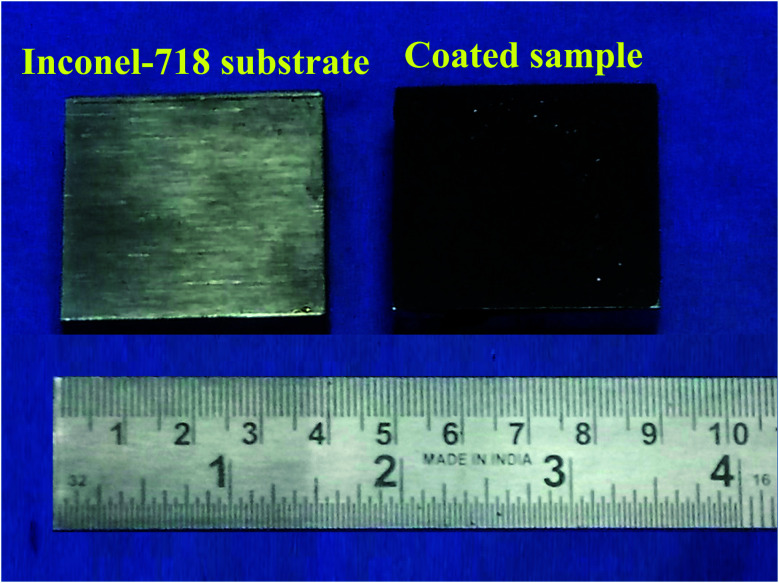
Macropicture of Inconel-718 substrate and coated samples.

**Fig. 3:**
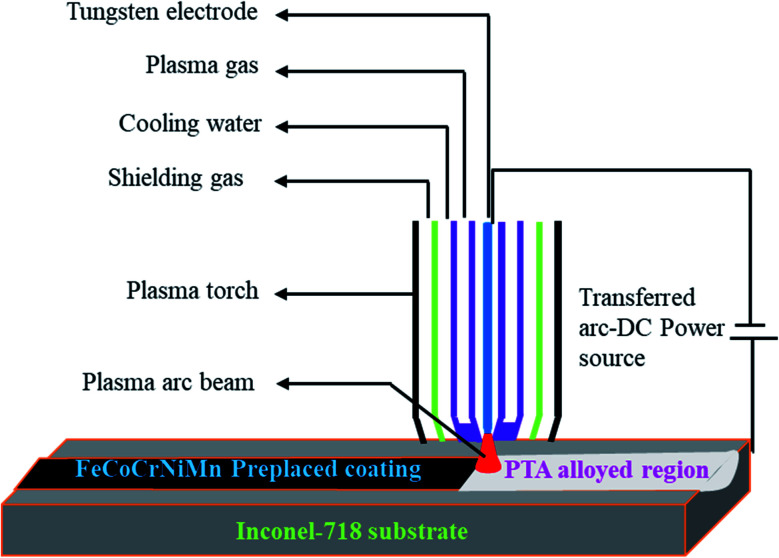
Schematic representation of PTA alloying process.

Deionized water was heated for 20 min continuously, and polyvinyl alcohol (PVA) was added in the ratio of 85 : 15. The PVA-added deionized water was further heated to 260 °C for 30 min using a hot plate, and was stirred magnetically to dissolve completely. Then, the ball-milled alloy powder FeCoCrNiMn was mixed with the PVA solution, and stirred for 90 min to form a colourless thick paste. The prepared thick paste, which was in the form of an alloy powder, and PVA were preplaced together on the substrate for the thickness of 200 μm, and kept in the fume cupboard for 48 hours to dry the preplaced layer. The macropicture of the preplaced specimen and Inconel-718 substrate are shown in Fig. 2. The prepared samples were surface-alloyed using a PTA welding machine with tungsten thorium electrode and argon as a shielding gas, as shown in Fig. 3. Table 2 shows the PTA process parameters used in the present experimentation.

**Table 2:** Optimized PTA process parameter

Parameters	Value
Arc current (A)	107
Arc voltage (V)	27.9
Scanning speed (mm s^−1^)	1
Type of gas flow rate (lpm)	Argon
Shield gas (lpm)	9.5
Center gas (lpm)	2.6
Defection angle (deg.)	90°
Overlapping (%)	25
Interpass (deg.)	300°
Tungsten rod	2% thoriated

### 2.3 Characterization

The phase analysis was performed on a PTA-alloyed specimen and substrate (D/MAX/2200/PC Ultima IV, Rigaku, Japan) to reveal the phase formation. Then, the PTA alloyed samples were cross-sectioned and polished with different grades of emery sheets to remove the oxide and irregularities. A final polishing was carried out with diamond paste with soft cloth and etched with Kallings reagent [5 g CuCl_2_; 100 ml HCl; 100 ml ethanol] for a few seconds, and washed in running water to reveal the microstructure. The structure of the alloyed samples was investigated using optical microscopy (OM) (BX41M-LED, Olympus, Japan) and Field Emission-Scanning Electron Microscopy (FESEM) (Sigma Gemini Column, Carl Zeiss, USA). The nanohardness test was carried out on the alloyed, interface and substrate regions with the force of 2000 μN using TI 980 TriboIndenter. Samples with dimensions of 08 × 08 × 15 mm were prepared for the wear test. Hardened stainless steel was selected as the counter material. Dry sliding wear test was carried out on a pin-on-disc wear monitor (TR-20LE-PHM 400-CHM 500, Ducom Instruments, India) with two different parameters, namely, loads of 30 N, sliding velocity of 1.1 m s^−1^ and load of 50 N and 0.9 m s^−1^ sliding velocity, with a constant sliding distance of 2500 m. Then, mass losses were calculated by subtracting the initial masses from the final masses of the wear tested samples. The wear rate and CoF are calculated using the eqn (1) and (2).1
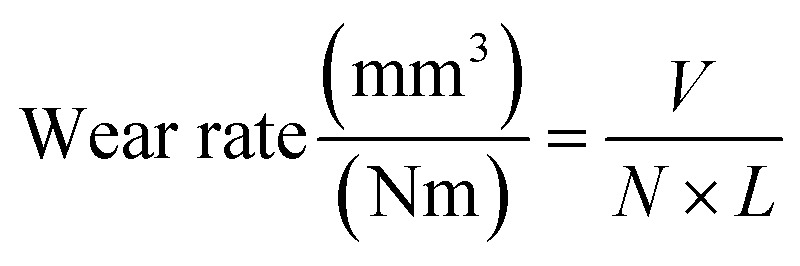
2
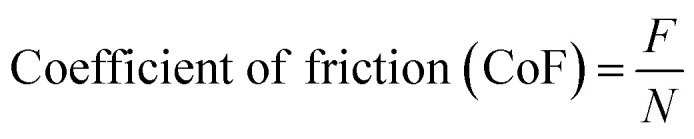
where *V* is the wear volume (mm^3^), *N* is the applied load (N), *L* is the sliding distance (m), and *F* is the friction force (N). Furthermore, worn-out surfaces were analysed through FESEM to study their various wear mechanisms. The surface roughness of the worn-out samples was measured using a whitelight interferometer (MFT-5000, Rtec Instruments, USA).

## 3. Results and discussion

### 3.1 Phase analysis

**Fig. 4:**
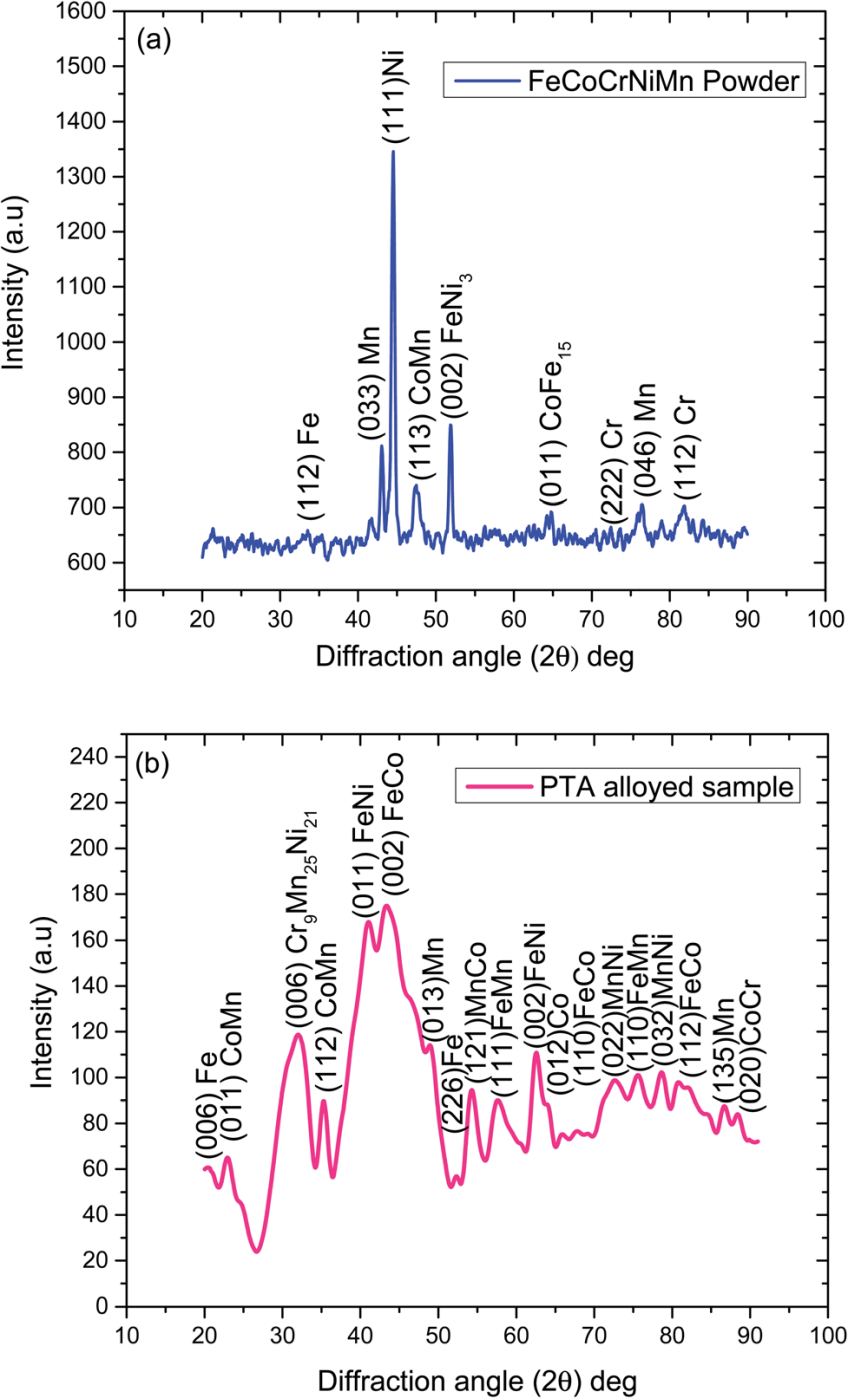
XRD analysis of: (a) FeCoCrNiMn powder, (b) PTA alloyed sample.

XRD analysis was performed on the ball-milled FeCoCrNiMn particles, and their corresponding peak locations were studied. The ball-milled powders induced frictional collision between the elements in the alloy system. This frictional collision resulted in the formation of some secondary phase particles. Notably, a few broadened peaks were observed at about 2*θ* = 40° to 55°. Moreover, it confirmed the presence of all alloying elements in the ball-milled powder. Some compound phases, such as CoMn, FeNi_3_ and CoFe_15_, were also traced in this XRD analysis. Fig. 4(a) shows the XRD spectrum of the powder materials.

Fig. 4(b) shows the XRD analysis of the PTA alloyed surface. During the PTA alloying process, the preplaced FeCoCrNiMn coating and the top surface of the Inconel-718 substrate were molten simultaneously. The molten pool of the preplaced coating, along with the dilution of top surface of the Inconel-718 substrate, was formed rapidly. The alloying elements Fe, Co, Cr, Ni and Mn in the preplaced coating were very rapidly dissolved in the molten pool. The molten pool that contained the alloyed elements were solidified after the forward movement of the plasma arc. The XRD pattern revealed that the FeCoCrNiMn-alloyed PTA region contained different phases, such as FCC, BCC and intermetallic.^19^ Most of the alloying elements were formed in the compound phase, which shows that the high specific energy density of the plasma transferred arc results in sufficient dissolution of alloying elements in the Inconel-718 substrate. It was found that the alloyed region included CoMn, CoCr, Cr_9_Mn_25_Ni_21_, FeNi, MnNi, FeCo, FeMn and MnCo. Miller indices of (*h*,*k*,*l*) values were identified by X'Pert high score software. In the BCC phase, the reflection is allowed only when *h* + *k* + *l* is an even value.^20^ In the FCC phase, reflection is allowed only when *h*, *k* and *l* are all odd or all even (unmixed). Based on these rules, (011) CoMn, (110) FeCo, (011) FeNi, (013) Mn, (112) CoMn, (121) MnCo, (110) FeMn, (112) FeCo, and FeCo were identified as the BCC phase. (111) FeMn and (135) Mn were identified as the FCC phase. (006) Fe, (022) MnNi, (006) Cr_9_Mn_25_Ni_21_, (002) FeCo, (226) Fe, (002) FeNi, and (020) CoCr all obey both the rules. Hence, these phases are concluded as either BCC or FCC phases. (032) MnNi, (135) Mn and (012) Co are the phases that do not obey any of the above rules. Consequently, these phases are concluded as intermetallic phases.

The number of peaks drastically increased in the alloyed region due to more lattice distortion. Moreover, there was a shift in the phases in the alloyed region owing to the formation of compound phases. The diffraction peaks widened at the diffraction angle of 32° and 40° due to strong formation of the BCC phase. Most of the alloyed region occupied the BCC phase. There was some elementary Co, Fe and Mn phase in the alloying region after the performance of the FeCoCrNiMn PTA alloyed process. The Co element easily dissolves in the Inconel-718 substrate as the atomic radii of Ni and Co are close to each other. As a result, Co with Ni compounds was not observed in the alloyed region.^21^

### 3.2 Microstructure analysis

The ball-milled mechanically alloyed powder contained particles in the range of 3 to 10 μm. The external view of the ball-milled powder after 12 h is shown in Fig. 1. It mixes the different particle size powders through the mechanical alloying process. Each particle underwent multiple plastic deformations during the ball-milling process. The microstructure and morphology of the alloy particles were mixed thoroughly due to the leading role of friction and shear deformation. The results of the processing alloy powder in this ball mill process were reducing the particle size, as well as the blending of particles into new phases. Repeated processes, such as welding, fracturing and rewelding of powder particles, were involved during this blending process. Changes of the kinetic energy of the milling powder were inducted by the total amount of energy transferred to the alloy powder particles from balls during the milling process.

The reduction in the size of the particles depended on the ball size, rotational speed of the drum, alloying elements and duration of the milling time. In this study, the ball size, rotational speed of the drum and alloying elements were kept constant and the milling time was only considered up to the steady state level. The ball milling process was performed for up to 12 h. Even upon increasing the milling time beyond 12 h, there was no reduction in the size of the particles as the particles reduced the maximum exitances under this combination of the milling setups. If the ball size is reduced, then the particles size may be reduced. However, based on the availability of the facility, this milling process was restricted with this ball milling specification. Moreover, there was variation in the particles size due to variation in the hardness of the particles. The hardness of the Cr and Co elements were higher than that of the other alloying elements. The dispersal of Cr and Co powders did not exhibit any improvement with the increase of the milling time to 12 h because it was difficult to crush this element in these powders. Furthermore, we were unable to make them small due to their high hardness, causing heterogenization. As a result, the uneven distribution of the particles appeared in the FESEM image of the ball-milled powder (Fig. 1).

**Fig. 5:**
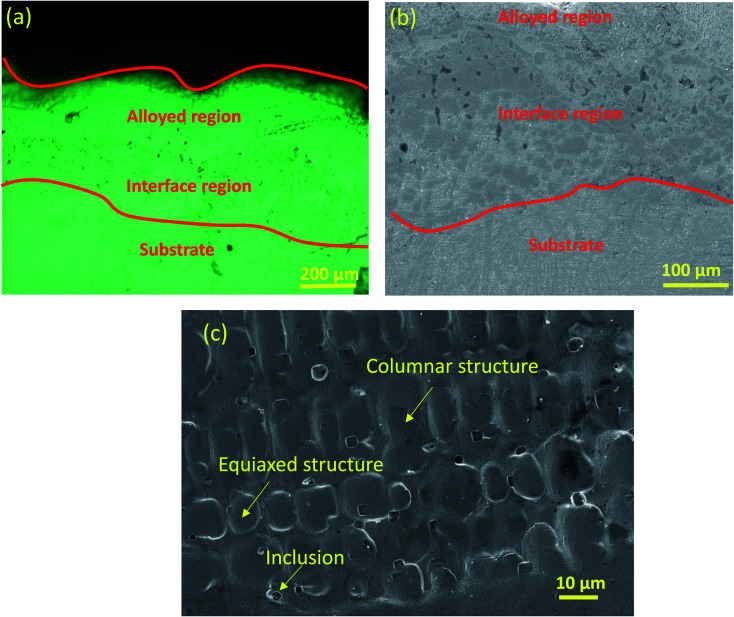
(a) Cross-sectional view of PTA alloyed region with their thickness, (b) FESEM image of interface region, (c) alloyed center region with showing columnar and equiaxed structure.

The EDS analysis shows that the ball-milled powders contain all essential chemical elements, such as Fe, Co, Cr, Ni and Mn. The cross-section of the PTA-alloyed region is shown in Fig. 5. Fig. 5(a) was taken by using an optical micrograph, whereas Fig. 5(b and c) were taken by using field emission-scanning electron microscopy. The cross-section of the PTA-treated sample contained the PTA-alloyed region, interface region and substrate region. The thickness of the alloyed region was measured along the line perpendicular to the direction of the movement of the PTA torch. The measured thickness of the alloyed region was 200 μm, which is clearly visible in Fig. 5(a). Fig. 5(b) shows the interface region of the PTA-alloyed region and substrate region. This interface region contained both alloying elements and substrate material. Based on the EDS analysis, the dark gray color areas in Fig. 5(b) were rich in Co and Mn elements that are dispersed non-uniformly with irregular shapes. The gray color areas are rich in Ni, Fe and Cr elements.

The PTA alloying machine has a tungsten electrode (cathode electrode), which was connected to a negative terminal. The work-piece acted as an anode electrode that connected to the positive terminal. The gas mixture was supplied to the PTA welding torch nozzle that produced an arc between the electrodes. The gas molecules collided with the PTA arc, which resulted in disassociating the gas molecules into atoms. Furthermore, these atoms divided into ions and electrons due to the high-temperature arc. It resulted in the formation of electrically charged ions. During this ionization process, more heat energy was liberated between the specimen and the welding torch. This high energy plasma moved with high velocity towards the FeCoCrNiMn-coated surface. The PTA alloying process limited the grain growth due to the rapid solidification process, and refined the size of the grain in the range of 2–10 μm. The gas bubbles in the molten region were relieved from the PTA-alloyed region due the influence of the buoyancy effects. Moreover, the directional solidification and rapid cooling rate reduced the formation of porosity in the alloyed region. The PTA alloy has needle-like dendrites that have a width of 5–10 μm and length of 10–15 μm. These columnar grains were grown in the longitudinal direction, which was perpendicular to the movement of the molten pool in the PTA FeCoCrNiMn-alloyed region due to the high thermal gradient region.

The high metallurgical bonding between the alloy particles and Inconel-718 substrate can be observed. No cracks were observed in the PTA-alloyed region. Moreover, a porous free structure was viewed in the alloyed region, which reveals that the PTA alloying process can be effectively used to perform the alloying process. Fig. 5(c) shows the microstructure of the central portion of the structure alloyed by the PTA process. The microstructure of the alloyed region shows the numerous micro impressions in the columnar and equiaxed shapes. These different-shaped micro impressions are the solidified patterns due to the different solidification nature of the alloying elements in the FeCoCrNiMn alloy powder composition. In addition to that, the presence of a few inclusions is observed throughout the microstructure. Uneven solidification traps the melts in the matrix material as an inclusion. The micro impressions are formed in layers in the matrix. Besides, there is no observation of a distinct variation in the microstructure phases. The average grain sizes are measured as ∼3 μm. This extreme grain refinement has resulted in the rapid solidification of a molten pool during the PTA treatment. This indicates that proper metallurgical alloying occurred between the elements added in the PTA FeCoCrNiMn-alloyed region.

**Fig. 6:**
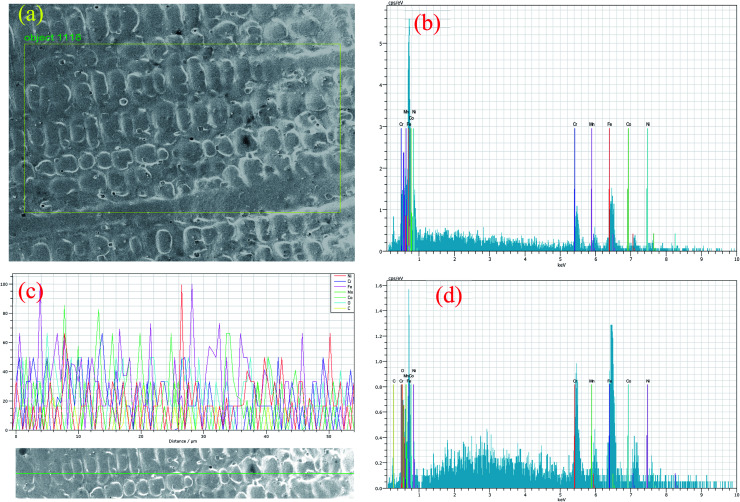
(a) FESEM images of PTA alloyed region, corresponding EDS graph at: (b) area analysis, (c) line analysis and (d) point analysis.

Fig. 6(a–d) shows the EDS analysis of the PTA-alloyed region. The obtained EDS analysis indicates the presence of all alloying elements after the PTA process [Fig. 6(b)]. Table 3 lists the presence of elements obtained in the PTA-alloyed region. The elements Fe, Cr and Ni are observed as major elements with atomic wt% of 32.48, 25.59 and 16.48, respectively. The remaining positions are occupied by Mn and Co. Therefore, it is confirmed that the Fe element constitutes the matrix material for this PTA-alloyed system. The EDS analysis has proved that the diffusion of the elements of the substrate has never occurred in the central zone of the surface-alloyed region. The heat input by PTA has been majorly utilized for the melting of the alloying elements, and very meagerly utilized to melt the substrate for bonding. Therefore, the diffusion of the elements of the substrate was restricted. Fig. 6 (c and d) shows the EDS result of the interface region and substrate. This line and point scan indicate that the diffusion of major alloying elements, like Ni, Cr, Co, Fe and Mn, has occurred on either side; especially, diffusion mainly occurred on the substrate side. The EDS analysis clearly indicates that the surface treatment completely protects the Inconel-718 substrate, and no trace of the substrate chemical elements is found in the treated layer.

**Table 3:** EDS analysis results of PTA alloyed surface at different region

EDS analysis results (in atomic wt%)	Fe	Co	Cr	Ni	Mn
Area analysis [Fig. 6(b)]	32.48	12.33	25.59	16.48	13.12
Point analysis [Fig. 6(d)]	29.21	14.56	23.48	22.73	10.02

### 3.3 Nanoindentation examination

**Fig. 7:**
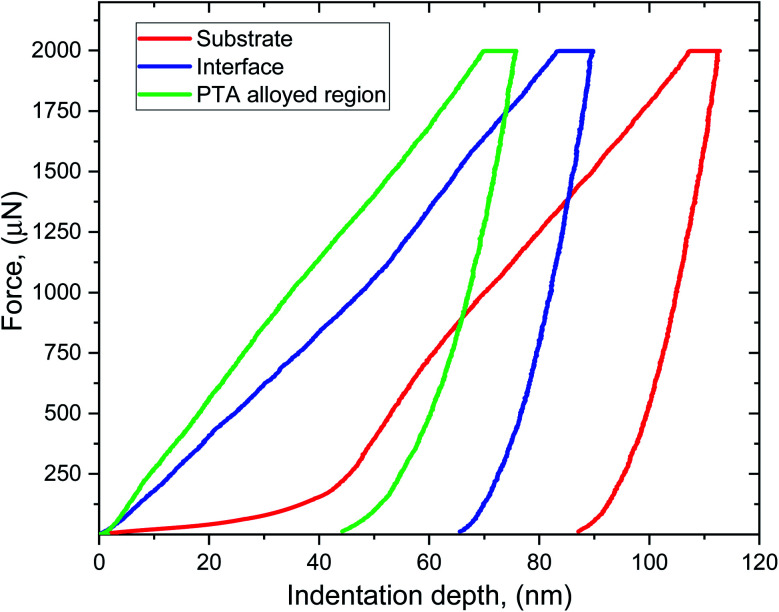
Nanohardness profile of PTA alloyed, interface, substrate region.

Hardness is one of the important properties of the material performance, as it is closely associated with the wear resistance of the specimen. Fig. 7 shows the nanohardness profile of the substrate, interface and PTA-alloyed surface. The nanohardness of the substrate, interface and PTA-alloyed layer were 2.921 GPa, 4.051 GPa and 5.157 GPa, respectively. The main factors that affected the nanohardness of PTA alloying included the element contents and grain size of the alloyed region. The PTA treatment melted the alloy powders on the substrate surface, and rapid solidification occurred during the raster scanning of PTA. Owing to the rapid solidification process of the PTA alloyed region, the grains were refined as fine particles. This rapid solidification initiated the hard phase compounds in the matrix surface.^22^ During the measurement of nanohardness, an external force was applied on the grains that induced the plastic deformation of the grains. The PTA-alloyed region had fine grains that dispersed the plastic deformation to more grains instead of single grains. As a result, stress acting on each grain was reduced that led to an increase in the nanohardness of the alloyed region. Besides, the Co element increased the solubility of the Cr element in the Inconel-718 substrate. The Co element in the alloyed and interface regions improved the high temperature property. Consequently, the nanohardness of the alloying region increased. The nanohardness of the interface region decreased most notably owing to the lack of a hard phase and the alloying elements. Moreover, the solid solution strengthening of the Fe, Co, Cr, Ni and Mn alloying elements increased the hardness of the alloyed region.

Different compounds were formed in the PTA-alloyed region, such as NiFe, CoMn, Cr_9_Mn_25_Ni_21_, MnNi, FeCo, FeMn and MnCo. Among these compounds, most of them have the BCC phase that has less slip distance compared to that of other phases. It resulted in the reduction of the movement of elements during the hardness measurement compared to that of the Inconel-718 substrate. The solid solution strengthening of the FeNi compound increased the hardness of the alloyed region, as the diameter of Ni and Fe were 0.125 nm and 0.124 nm, respectively. The atomic radii of the Ni and Fe elements are very close to each other. Both Fe atoms and Ni atoms interchange with each other, and formed the high strength solid solution. When the external force acted on the grain, the plastic deformation was dispersed into more grains. It resulted in a small uniform plastic deformation on each grain due to less stress concentration. Hence, the hardness of the NiFe PTA alloy coating was obviously improved owing to the combined effect of fine grain strengthening and solid solution strengthening. Jinku Yu *et al.*^23^ investigated the hardness of the NiFe coating, and found that the hardness of the NiFe coating enhanced the hardness compared to the Ni-based substrate due to fine grains formation and solid solution strengthening. Other compounds, such as CoMn, FeCo, FeMn and MnCo, possessed BCC phases that have higher hardness than other forms of phases.

The atomic sizes of the alloying elements other than the Ni elements were much larger. The variation in the atomic radius led to a large lattice distortion that increased the resistance of the alloyed region and reduced the dislocation motion. It resulted in the diminishing of the local stress concentration at the alloyed region.

### 3.4 Wear and friction behaviour

**Fig. 8:**
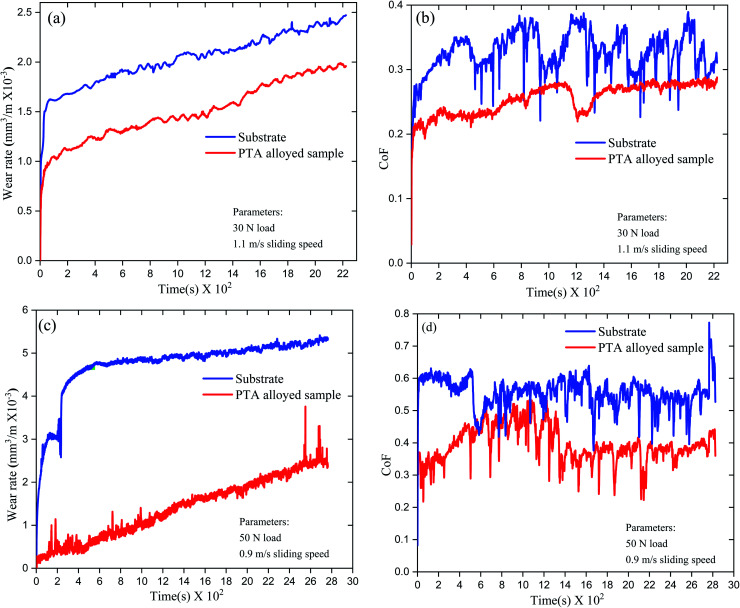
(a and b) The obtained wear rate and co-efficient of friction graph at the applied load of 30 N, (c and d) wear rate and co-efficient of friction graph at the applied load of 50 N.

Fig. 8(a and c) show the wear rates of substrate and PTA alloyed samples at different input parameter conditions. The wear rate of the substrate specimen at 30 N applied load is 2.45 × 10^−3^ mm^3^ m^−1^ and for the PTA specimen, it is 1.79 × 10^−3^ mm^3^ m^−1^. Similarly, the wear rate of the substrate specimen at 50 N is 5.38 × 10^−3^ mm^3^ m^−1^ and for the PTA sample, it is 2.29 × 10^−3^ mm^3^ m^−1^. Comparison of the wear rates of the substrate and PTA specimen revealed that the PTA surfaces showed significantly improved wear resistance. Moreover, an increase in the applied load increased the wear rate in both substrate and PTA surfaces. The untreated surface tested at 50 N of applied load showed higher wear rate than the rest of the samples.

Wear losses commonly occur at a softer surface when it contacts with the counter hard surface. The metallic materials with different contact regimes experience various frictional forces due to the intensity of the surface asperities and surface hardness. The intensity of the asperities is high in the untreated surface. Cold welding of the surface asperities between the counter material and substrate happened during the wear test loading. The weaker asperities usually present in the substrate surface got fractured during the relative sliding. When the test load increased, bonding between the asperities deepened and the amount of fracture escalated during the relative sliding. Therefore, wear losses in the 50 N test loading were higher than 30 N in both untreated and treated surfaces. Moreover, the surface hardness plays a significant role in minimizing the wear losses. Higher surface hardness increases the fracture toughness of asperities, which minimizes the wear losses due to friction. Surface treatment of Inconel-718 by the PTA alloying process improved the surface hardness significantly.^24^ The presence of intermetallic particles increased the hardness and arrested the crack propagation. As noted earlier, the intensity of the surface asperities affects the wear losses. The intensity of the asperities is higher in the untreated surface than the treated surface.

Fig. 8(b) shows the CoF of the untreated and treated samples tested at an applied load of 30 N. The CoF of the untreated sample shows an increasing trend from 0 to 0.4 at 400 s. Then, a sudden drop has occurred at 500 s, gradually increasing up to 1200 s. A similar drop in CoF is observed at 1200 s, 1400 s and 1700 s. The maximum drop of 0.25 CoF is observed at 1000 s. The initial steep increase in CoF resulted from the resistance of asperities for deformation. The frictional force minimizes when the asperities started deforming. However, the fractured asperities accumulated in the sliding path and acted as an obstacle for the forward sliding. Moreover, the fractured asperities cold-welded with the existing asperities, and formed a plasticized layer. The effect of accumulation of fractured asperities and formation of thin layer increased the frictional force to deform them. Then, the reduction of frictional force happened due to less resistance for deformation. This cycle continued until the completion of the wear test. The lower surface hardness and higher ductility are the cause for this variation of CoF in the untreated sample.

Fig. 8(b) also shows the CoF of the treated specimen tested at an applied load of 30 N. It can be observed that the CoF shows minimum fluctuations compared with the untreated sample. CoF increases up to 0.28 at 250 s. Then, a slight drop to 0.22 occurred after 250 s to 750 s. A steady increase in CoF up to 0.26 happened at 1250 s. A sharp drop in CoF occurred after 1200 s, and was retained up to 1500 s. After 1500 s, CoF has recovered and stabilized until the end of the test. Compared to the untreated sample, the fluctuations in the frictional forces are low in the treated sample. Initially, frictional forces were low during the test. However, when the test time increased, the frictional forces increased with time and attained a maximum around 1000 s to 1250 s. The PTA-alloyed surface improved the surface hardness significantly. Thus, a minimum deformation of the asperities occurred during the initial stages of the test. However, accumulation of the deformed asperities along the sliding path increased the frictional force for further deformation. The sudden decline of frictional forces was due to the bulk deformation of the accumulated thin layer of the deformed asperities. Then, the frictional forces have been regained and stabilized.

Fig. 8(d) shows the CoF of untreated samples tested at an applied load of 50 N. During the starting of the test, CoF of the untreated sample increased to 0.62 and was maintained with minor fluctuations up to 300 s. After 500 s, a sharp decline in the frictional force has been observed and major fluctuations happened over the testing. Absence of the cyclic variation in CoF was observed in 50 N loading condition compared with 30 N loading. The irregular high intensity asperities in the untreated surface have cold-welded with the counter material, and increased the frictional force at the initial stage. When the applied load exceeded the frictional resistance of the cold-welded asperities, a huge deformation occurred. Thus, it reduced the frictional forces suddenly. However, recovery of CoF happened due to the cold-welding of the asperities again, and the increased applied load lowered the frictional forces.

Fig. 8(d) shows the CoF of the treated specimen at 50 N applied load. It can be observed that the treated specimen showed lower CoF than the untreated sample. In addition to that, wide fluctuations in the frictional forces are observed. The frictional forces are increasing over the testing time up to 0.45 at 500 s. A sharp decline in the frictional force occurred at 500 s. Then, it slightly increased to 0.50. This decline and recovery of CoF was observed throughout the test period. However, after 1500 s testing period, CoF reduced from its maximum range, and there were continuous fluctuations until the end. The surface-alloyed Inconel-718 improved the hardness through various particle elements. The minimum deformation of the surface asperities happened during the wear testing due to the increased hardness. The presence of intermetallic compounds and improved hardness prevented the crack propagation. The fluctuations in the CoF indicate the variations in the deformation behaviour of asperities. Decline of CoF after 1500 s of test time shows the stabilization of the surface with minimum deformation of the surface asperities. Even at increased applied load, due to increased surface hardness, the minimum deformation of asperities resulted with a reduction of the frictional forces.

### 3.5 Wear mechanism

**Fig. 9:**
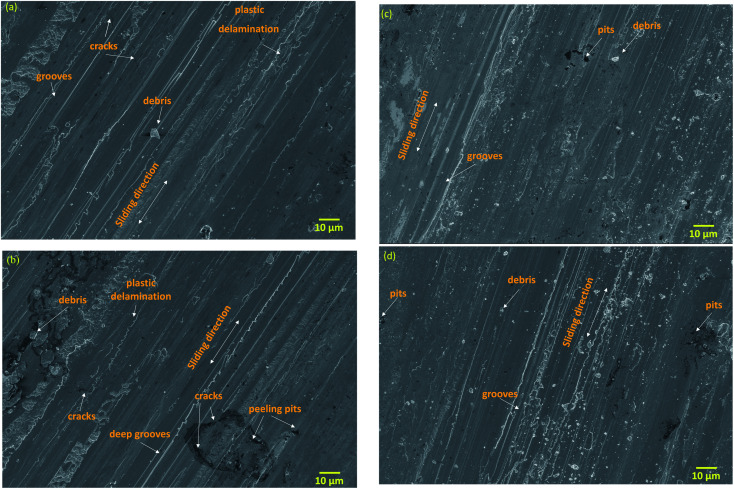
Worn-out surfaces: (a and b) substrate at 30 N and 50 N load, respectively. (c and d) PTA alloyed specimen at 30 N and 50 N load, respectively.

Fig. 9(a) shows the worn-out surfaces of the substrate specimen at an applied load of 30 N. Fig. 9(a) shows the combination of adhesive and abrasive wear losses in the worn-out substrate surface. Lower hardness of the substrate accelerated the adhesive wear losses. Adhesive tendency in the substrate material varied with the nature of asperities in it. Furthermore, it is clear that a few areas in the substrate have experienced abrasive wear losses. The compressive stresses were induced in the substrate surface due to the applied load. Due to the lower surface hardness, this compressive stress initiated microcracks and plastic deformation. Fig. 9(b) shows the worn-out surface of the substrate at 50 N load condition. The worn-out surface indicates severe abrasive and adhesive wears. Along with the abrasive and adhesive wear, microcracks and plastic deformations are also observed. The adhesive wear dominated the worn-out surface of the substrate. Higher adhesive wear was attributed to the lower hardness of the substrate surface. Adhesive tendency of chemical elements like Al and Ni with the counter material during sliding increased the adhesive wear in the substrate material. Since adhesive wear was the dominant wear mechanism in the substrate, a few abrasive grooves were observed along with the adhesively worn-out areas. These abrasive losses occurred due to repetitive contact of the counter material with the adhesively bonded substrate on them.

Fig. 9(c) shows the worn-out surface of the treated surface at 30 N load condition. The worn-out surface revealed that wear losses were minimal and dominated by abrasive wear losses. A few narrow and shallow abrasive grooves were present in the worn-out surface. The absence of or a negligible amount of adhesive wear losses was observed. Improved hardness of the treated surface restricted the adhesive wear losses and plastic deformation due to compressive stresses. Fig. 9(d) shows the worn-out surface of the PTA alloyed specimen at 50 N applied load. The worn-out surface shows the abrasive grooves. The wear type of the PTA alloyed specimen consisted of mild abrasion with oxidative wear. Different oxides were formed during the wear test and protected the PTA-alloyed surface. The results have shown that the heterogeneous oxidation film forms on the worn-out surface. The oxidation layer on the PTA-alloyed surface of the FeCoCrNiMn coating was dense, and protected the alloyed specimen well. Hence, surface treatment has effectively protected the substrate, even at an increased applied load.^25^

**Fig. 10:**
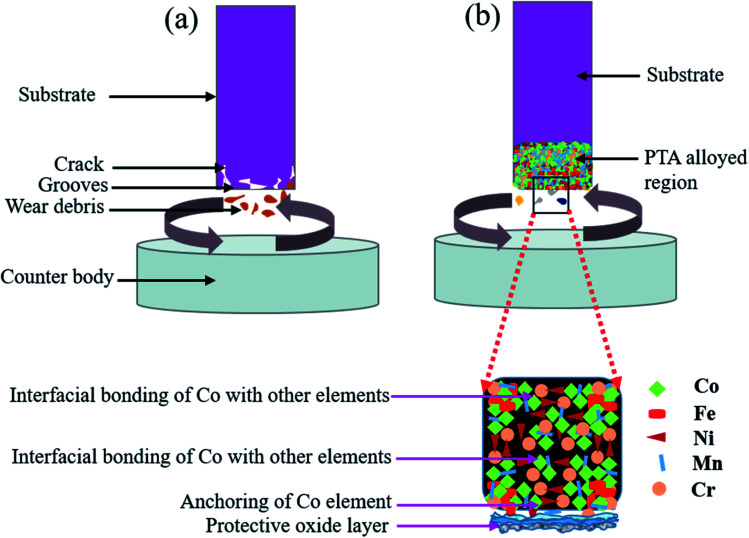
Schematic representation of wear mechanism: (a) substrate showing with crack, deformation and deep grooves, (b) PTA alloyed specimen showing with mild and small grooves.

A schematic representation of the wear mechanism in the Inconel-718 substrate and the PTA alloyed specimen is shown in Fig. 10. During the wear test, the formation of thick oxide layers on the worn-out surface was followed by frictional heat generation. However, the bearing capacity of the Inconel-718 substrate to the oxide layers was weak due to the low density. As a result, the oxide layer rubbed easily under irregular stress condition, and the fresh substrate surface was exposed. Consequently, the delaminate oxide layers, which have high hardness that turn into be harder abrasive particles, aggravated the wear of the Inconel-718 substrate and led to a reduction of the wear resistance and increased the CoF, which is shown in Fig. 10(a). In the PTA-alloyed worn-out surface, the number of grooves decreased and the pits became relatively smaller. In addition to that, the worn-out surface became smoother and fine, which is represented in Fig. 10(b). The important phase of the alloyed region included α-Fe, γ-Fe, the carbide (Fe, Cr)_3_C, intermetallic compounds FeNi, CoCr, CoMn, CrFeNi and oxides that have higher hardness. It served as supporting particles that reduced the contact area between the tribo-pair. Therefore, the CoF was continued at the lower level.

**Fig. 11:**
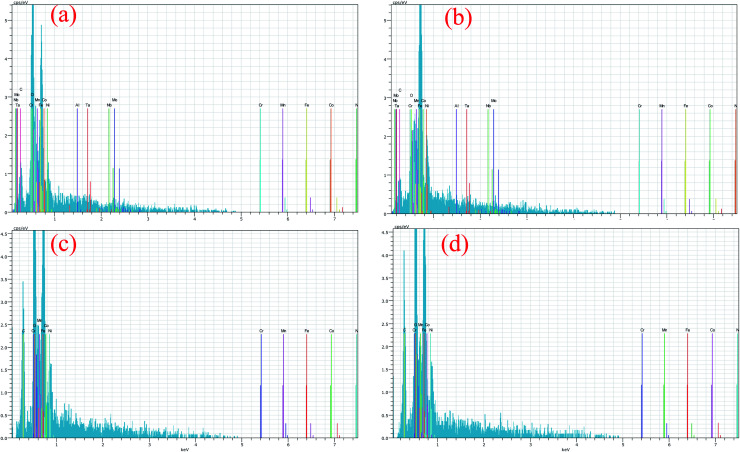
EDS analysis of worn-out surfaces: (a and b) substrate at 30 N and 50 N load, respectively. (c and d) PTA alloyed specimen at 30 N and 50 N load, respectively.

The EDS results [Fig. 11(a and b)] of the worn-out surface indicate that the reduction of the Al and Ni elements in the substrate is advocating the concept of adhesive wear loss due to those elements. Table 4 shows the detailed EDS results with their elemental percentages. While increasing the applied load, the adhesiveness of the substrate surface with the counter material increased and deformed drastically during sliding. The EDS [Fig. 11(c and d)] results proved that the worn-out surface contains the FeCoCrNiMn chemical elements with the absence of the substrate chemical elements. No traces of substrate chemical elements were found on the worn-out surfaces. Moreover, the presence of oxides in the worn-out surface acted as a barrier and protected the treated surface from an aggressive environment. This indicates that the PTA alloyed treatment significantly improved the surface protection.

**Table 4:** EDS analysis results of worn-out surface of substrate and PTA alloyed samples

EDS analysis results (in atomic wt%)	Fe	Co	Cr	Ni	Mn	O	Nb	Mo	Al
Substrate sample at 30 N load [Fig. 10(a)]	21.58	0.83	16.93	42.71	0.40	10.26	3.52	3.22	0.55
Substrate sample at 50 N load [Fig. 10(b)]	22.94	0.63	15.47	45.12	0.64	10.93	2.82	1.12	0.33
PTA sample at 30 N load [Fig. 10(c)]	18.46	12.47	11.75	10.34	14.04	32.94	—	—	—
PTA sample at 50 N load [Fig. 10(d)]	20.71	13.85	11.97	9.56	10.82	33.09	—	—	—

### 3.6 Influence of randomly distributed alloying particles and oxide layer formation

During the wear test, the temperature of the contact surface was increased and the formation of an oxide layer was also increased under high contact load. These oxide layers were stacked continuously, and increased the relative density of the oxide layers. Considering the chemical compositions of the worn-out surface, it was observed that the presence of oxygen on the worn-out surface of the PTA-alloyed region showed relatively higher value. This is owing to a reduction of fragmentation and elimination of the exfoliation of oxide layers. It reveals that the primary wear type is mild abrasive wear, accompanied by slight oxidative wear. The oxide layer formed during this wear test process remained stable and undamaged. Oxygen reacted with the surface alloying elements, and formed different oxides, such as CoO, Cr_2_O_3_, MnO_2_, Mn_2_O_3_, Mn_3_O_4_, FeO and Fe_2_O_3_. Based on the EDS result (Fig. 11(c and d)), the presence of a significant amount of oxygen on the worn-out surface of the PTA alloyed surface was realised. The percentage of oxygen present in the PTA-alloyed region was 32.94% and 33.05% at 30 N and 50 N applied load, respectively. Moreover, a greater amount of oxygen was present on the surface compared to that of the worn-out surface of the substrate. For the wear rate at 30 N and 50 N load conditions, the PTA alloyed specimen had less value than of the substrate. The elements, such as Fe, Mn, Cr and Co, had an affinity to react with oxygen during the wear test and reduced the wear rate by acting as a barrier between the matting surface.^26–32^ These dense oxidation films covered the wear surface, and enhanced the wear resistance of the alloyed region. Shin J. H. *et al.*^26^ stated that the hardness of the Fe_2_O_3_ oxide layer was around 1000 HV. Hence, the oxide layers were developed as protective layers from the aggressive wear between the matting surface. Moreover, it avoided the direct contact of the PTA alloyed region to the counterpart that stepped down the wear rate.

The alloying elements influenced the formation of the oxide layer that reduced the crater formation, and resulted in the reduction of the wear rate and CoF. The Co element has acted as a binder and increased the interfacial bonding with other alloying elements.^27^ Alloying elements were not peeled off from the alloyed surface, owing to the anchoring effect of the Co element. As a result, delamination of alloying elements from the PTA tribo-surface was reduced. Moreover, the Co content increased the stronger interaction among the alloying elements. Thus, the deterioration of the friction properties was achieved. However, some of the oxides were weakened and were lost shortly. Compared to the Co elements, the Cr element has oxides Cr_2_O_3_ that enhanced the formation of the transient oxidation of chromium in the place of cobalt.^28^ However, the Cr oxides were lost continuously for the PTA-alloyed region at the initial stage of the wear test. The depletion in the Cr element led to the production of a larger quantity of CoO, which has higher diffusional property than Cr oxide and was regained in the surface.^29^ Additionally, the worn-out surface contained high levels of Fe, Cr, Co, Ni, O and Mn. The results have shown that the heterogeneous oxidation film was formed on the worn-out surface. As a result, the formation of oxidation film changed significantly, and composite oxide files were formed that consisted of a small content of Cr_2_O_3_ and large content of Fe_2_O_3_, NiO and CoO oxides. This composite oxidation layer effectively prevented the delamination of the alloyed region during the friction process.

Different forms of Fe oxides, such as FeO, Fe_2_O and Fe_3_O_4_, were likely formed during the wear test. FeO has the lowest oxygen content, while Fe_2_O_3_ has the highest oxygen content. During the wear test, a high vacancy concentration, and highly mobile electrons and ions occurred. Furthermore, it oxidized the Mn_2_O_3_ and FeO to MnO_2_ and Fe_2_O_3_, respectively. It resulted in the formation of different lattice structures of the oxide layer, and retained the bonding between the layers and the PTA-alloyed region. It further stabilized the CoF and reduced the wear rate. The Fe ion and electrons reacted with the external atmospheric environment, and formed the FeO oxide layer.^30^3
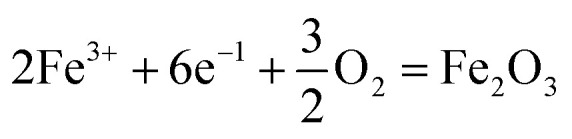

4 Fe^2+^ + 2e^−1^ + 4Fe_2_O_3_ = 3Fe_3_O_4_

Similarly, other oxides such as the Cr–Fe oxide, Mn oxide and Ni oxide were formed. The Mn element was oxidized, which led to the formation of MnO.^31^

5 2MnO + O_2_ = MnO_2_

6
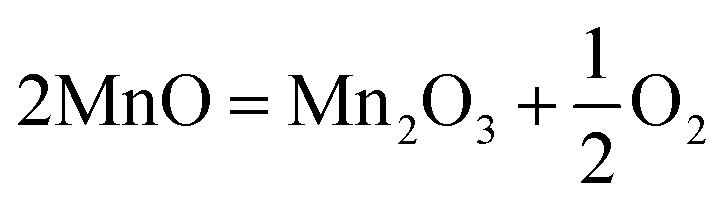


This Mn_2_O_3_ was deionized and formed Mn_3_O_4_. The Mn element was dissolved as a substitutional atom in α-Fe, and led to the distortion of the Fe lattice and produced the distortional energy.

7 Mn_3_O_4_ + Fe_2_O_3_ = MnFe_2_O_4_ + Mn_2_O_3_

The Mn elements increased the lattice parameter, which implies that the lattice distortion has resulted in the improvement of hardness. Hence, along with the compound form of the oxide formation with lattice distortion, the Mn element formed the hard phase and improved the hardness of the PTA-alloyed specimen. The solid solution strengthening of Mn led to increased wear resistance of the alloyed region. The element Mn increased the hardness by improving the higher relative density, solid solution strengthening and uniform distribution of hard particles. It resulted in the increase of the bearing capacity of the PTA-alloyed region compared to that of the oxide layers. These oxide layers acted as a barrier and protected the PTA-alloyed region from being ploughed by a hard phase abrasive material that reduced the possibility of the generation of abrasive grooves. Moreover, the high bearing capacity of the PTA-alloyed region increased the accumulation of the composite oxide layers, which resisted the contact of heat energy to the PTA-alloyed region. Besides, the formation of hard-phase chemical compounds, random distribution of alloying elements, and binding property of the Co element enhanced the bearing capability of the alloyed region and improved the wear resistance.

### 3.7 Worn-out roughness analysis

**Fig. 12:**
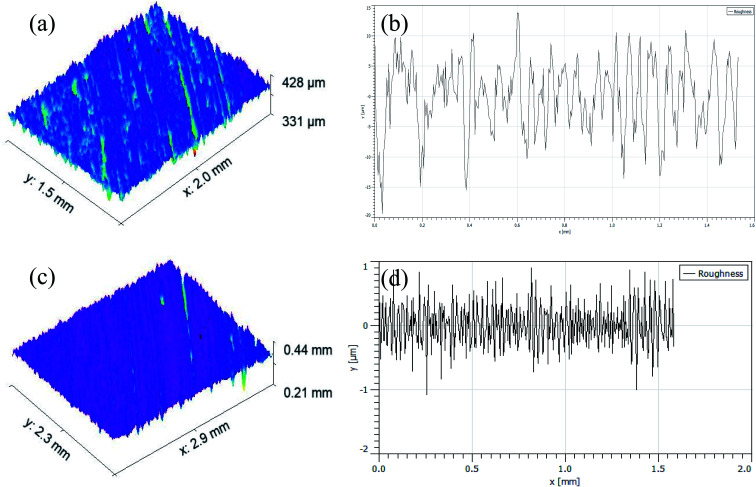
3D topography of worn-out surfaces and its roughness plot at 50 N load: (a and b) substrate, (c and d) PTA alloyed sample.

Fig. 12(a and b) shows the 3D topography, and the corresponding roughness profile of the worn-out untreated surface at 50 N load. The 3D topography shows numerous wear scar marks on the untreated surface, and these wear scar marks are deeper and wider. The 2D topography indicates that the surface undulations are very rough, and that deeper wear scars are present in regular intervals. The average surface roughness (*R*_a_) of the worn-out untreated surface is measured as 6.31 μm. Fig. 12(c and d) shows the 3D topography, and the corresponding roughness profile of the worn-out PTA-alloyed surface at 50 N load. The 3D topography shows the minimum scar marks and losses. In addition to that, the absence of deep grooves is observed in the 3D topography. The 2D topography indicates that the wear scar depths are shallow and lower than the untreated surface. The PTA-alloyed surface with FeCoCrNiMn particles has significantly improved the hardness and minimized the wear losses.^32^ The average surface roughness of the PTA alloyed worn-out surface has been measured as 4.81 μm. This reduced roughness indicates the effect of the surface alloying on the Inconel-718.

Inconel-718 is widely used as the disk, shaft, vanes and casing in the aircraft gas turbines. However, its poor tribological property restricted its applicability. There were different studies executed to examine the wear characteristics of the treated and untreated Inconel-718 alloy. Arash Maniee *et al.*^33^ investigated the wear resistance of Inconel 718, which was treated by plasma nitriding process. The wear test was carried out at 15 N load and 250 m sliding distance. The results revealed that the wear rate of the treated surface was less than the untreated surface. The present study was been performed at 50 N load and 2500 m sliding distance, which were 3- and 10-times higher load and sliding distance, respectively. However, the wear rate of the PTA-treated sample was slightly higher than that of the nitride-treated surface, which shows that the PTA-treated sample can be used for similar application. Anandakrishnan *et al.*^34^ studied the dry sliding wear behavior of the additively manufactured Inconel 718 material. The wear test was carried out at 10 to 30 N load and 2000 m sliding distance. Compared to it, the present study has been performed at higher load and sliding distance. However, the wear rate of the present study has been well matched with the above studies.

## 4. Conclusion

In summary, the mechanically ball-milled FeCoCrNiMn particles were surface-alloyed on the Inconel-718 superalloy using the PTA technique. The effect of the PTA surface alloying on the microstructure, nanoindentation and wear resistance has been investigated. Furthermore, the various wear mechanisms, influence of alloying elements, formation of oxide layers and worn-out surface roughness were analysed. The following conclusions were drawn from the obtained result.

• The XRD pattern revealed that the FeCoCrNiMn-alloyed PTA region contained the BCC phase of the CoMn, FeCo, FeNi, Mn, MnCo, FeMn, FeCo, FeCo compound, FCC phase of FeMn, and the Mn and intermetallic phases of MnNi, Mn and Co.

• The hardness of the Cr and Co elements were higher than that of the other alloying elements. The distribution of the Cr and Co powders did not exhibit any improvement with the increase of the milling time to 12 h.

• The PTA alloy has needle-like dendrites that has a width of 5–10 μm and length of 10–15 μm. These columnar grains were grown in the longitudinal direction, which was perpendicular to the movement of the molten pool in the PTA FeCoCrNiMn-alloyed region due to the high thermal gradient region.

• The microstructure of the alloyed region showed numerous micro impressions in the columnar and equiaxed shapes. The nanohardness of the substrate, interface and PTA-alloyed layer were 2.921 GPa, 4.051 GPa and 5.157 GPa, respectively. The solid solution strengthening of the FeNi compound increased the hardness of the alloyed region, as the diameter of Ni and Fe were 0.125 nm and 0.124 nm, respectively.

• The wear rate of the substrate specimen at 30 N applied load is 2.45 × 10^−3^ mm^3^ m^−1^ and for the PTA specimen, it is 1.79 × 10^−3^ mm^3^ m^−1^. Similarly, the wear rate of the substrate specimen at 50 N is 5.38 × 10^−3^ mm^3^ m^−1^ and for the PTA sample, it is 2.29 × 10^−3^ mm^3^ m^−1^.

• The CoF of the substrate specimen at both 30 N and 50 N load showed higher fluctuations than the PTA specimens. The higher intensity of asperities in the substrate specimen tend to cold-weld with the counter material surface, which resulted in more wear losses during sliding.

• Minimal fluctuation of CoF was observed in the PTA specimens due to improved surface hardness. Adhesive wear dominated the substrate, while abrasive wear was seen in the PTA-alloyed samples. Adhesive wear losses in the substrate were due to the lower surface hardness and ductility.

• PTA FeCoCrNiMn-alloyed Inconel-718 can replace the uncoated Inconel-718 disk, shaft, vanes and casing in the aircraft gas turbines due increase in the wear resistance and reduction in the frictional value.

## Author contributions

N. Jeyaprakash: conceptualization, methodology, investigation, data curation, writing – original draft preparation. Che-Hua Yang: formal analysis, visualization, supervision, resources, funding, writing – reviewing and editing. G. Prabu: conceptualization, methodology, data curation, writing – reviewing and editing. K. Ganesa Balamurugan: conceptualization, software, data curation.

## Conflicts of interest

The authors declare that they have no known competing financial interests or personal relationships.

## Supplementary Material
